# Retraction: Effects of thermal aging atmospheres on oxidation activity, element composition and microstructure of diesel soot particles

**DOI:** 10.1039/d4ra90118e

**Published:** 2024-10-04

**Authors:** He Huang, Zifei Ni, Wenkai Wang, Heng Chen

**Affiliations:** a School of Traffic Engineering, Nanjing Vocational University of Industry Technology Nanjing 210046 China 2018100903@niit.edu.cn +86-025-85864356

## Abstract

Retraction of ‘Effects of thermal aging atmospheres on oxidation activity, element composition and microstructure of diesel soot particles’ by He Huang *et al.*, *RSC Adv.*, 2023, **13**, 29975–29985, https://doi.org/10.1039/D3RA05340G.

The Royal Society of Chemistry, with the agreement of the authors, hereby wholly retracts this *RSC Advances* article due to similarities in the data with an article by C. Peng *et al.*^1^ and a Masters thesis by C. Peng^2^ that were not cited in the original article. Huang *et al.* have previously collaborated with C. Peng *et al.*,^1^ and errors during data processing led to inaccurate data being presented in this article.

Signed: He Huang, Zifei Ni, Wenkai Wang and Heng Chen

Date: 27th September 2024

Retraction endorsed by Laura Fisher, Executive Editor, *RSC Advances*
